# Arctic Mustard Flower Color Polymorphism Controlled by Petal-Specific Downregulation at the Threshold of the Anthocyanin Biosynthetic Pathway

**DOI:** 10.1371/journal.pone.0018230

**Published:** 2011-04-07

**Authors:** Cynthia A. Dick, Jason Buenrostro, Timothy Butler, Matthew L. Carlson, Daniel J. Kliebenstein, Justen B. Whittall

**Affiliations:** 1 Department of Biology, Santa Clara University, Santa Clara, California, United States of America; 2 Biological Sciences Department, University of Alaska, Anchorage, Alaska, United States of America; 3 Department of Plant Sciences, University of California Davis, Davis, California, United States of America; United States Department of Agriculture, Agricultural Research Service, United States of America

## Abstract

Intra- and interspecific variation in flower color is a hallmark of angiosperm diversity. The evolutionary forces underlying the variety of flower colors can be nearly as diverse as the colors themselves. In addition to pollinator preferences, non-pollinator agents of selection can have a major influence on the evolution of flower color polymorphisms, especially when the pigments in question are also expressed in vegetative tissues. In such cases, identifying the target(s) of selection starts with determining the biochemical and molecular basis for the flower color variation and examining any pleiotropic effects manifested in vegetative tissues. Herein, we describe a widespread purple-white flower color polymorphism in the mustard *Parrya nudicaulis* spanning Alaska. The frequency of white-flowered individuals increases with increasing growing-season temperature, consistent with the role of anthocyanin pigments in stress tolerance. White petals fail to produce the stress responsive flavonoid intermediates in the anthocyanin biosynthetic pathway (ABP), suggesting an early pathway blockage. Petal cDNA sequences did not reveal blockages in any of the eight enzyme-coding genes in white-flowered individuals, nor any color differentiating SNPs. A qRT-PCR analysis of white petals identified a 24-fold reduction in chalcone synthase (*CHS*) at the threshold of the ABP, but no change in *CHS* expression in leaves and sepals. This arctic species has avoided the deleterious effects associated with the loss of flavonoid intermediates in vegetative tissues by decoupling *CHS* expression in petals and leaves, yet the correlation of flower color and climate suggests that the loss of flavonoids in the petals alone may affect the tolerance of white-flowered individuals to colder environments.

## Introduction

The spectacular diversity of angiosperm flower colors is often attributed to the preferences of their pollinators [1]–[3], yet the role of non-pollinator agents of selection and genetic drift must also be considered. When pollinators show no preference for particular morphs in flower color polymorphic populations, the frequencies of color morphs may be the result of genetic drift. In fact, lack of pollinator preference in the flower color polymorphic *Linanthus parryae* provided the motivating example for Wright's shifting balance theory that relied on the absence of natural selection [4]. Subsequent studies have not detected pollinator preference in this example, yet the possibility of natural selection cannot be completely excluded since flower color frequency correlates with spring precipitation, suggesting a non-pollinator agent of selection [5]. A diversity of additional non-pollinator agents of selection have been reported to affect the frequency of flower color morphs in polymorphic populations, including a range of abiotic factors (UV-light, hot and cold temperatures) and some biotic agents of selection (pathogens and herbivores) [6]–[8]. Whether these non-pollinator agents are acting directly on petal color or on pleiotropic byproducts of the flower color variation manifested in vegetative tissues, remains unclear [9].

**Figure 1:**
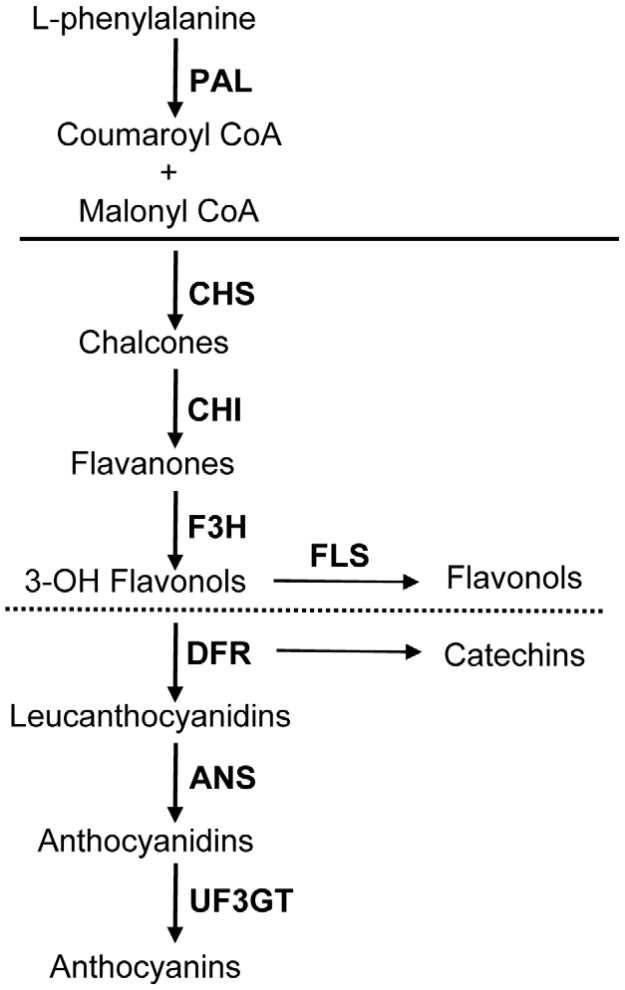
Genes involved in anthocyanin biosynthesis. The anthocyanin biosynthetic pathway consists of six core genes (below the solid line). We also examined a gene upstream from the ABP, phenylalanine ammonia lyase (*PAL*), and a side-branch enzyme that produces flavonols, flavonol synthase (*FLS*). The ABP can be divided into early and late halves based on the step that produces flavonols (dotted line). The core enzymes are abbreviated as follows: chalcone synthase (*CHS*), chalcone isomerase (*CHI*), flavanone-3-hydroxylase (*F3H*), dihydroflavonol 4-reductase (*DFR*), anthocyanidin synthase (*ANS*) and UDP glucose flavonoid 3-O-glucosyltransferase (*UF3GT*).

The most common and conspicuous flower color polymorphism among angiosperms is the loss of floral anthocyanins causing pigmented flowers to become white. Although subtle flower color variation can be caused by changes in epidermal cell shape, vacuolar pH, or the presence of cofactors [10], the shift from pigmented to white flowers requires a complete blockage of the six-step anthocyanin biosynthetic pathway (ABP; Figure 1) [11]. The ABP can be blocked by mutations leading to dysfunctional enzymes or through the downregulation of those enzymes. Anthocyanin pigments are also expressed in sepals, stems, leaves and even roots where they can be crucial to a plant's abiotic and biotic stress response [12]. Vegetative anthocyanins are critical for plant survival and reproduction. Thus, the loss of floral anthocyanins may be constrained by pleiotropic effects manifested in vegetative tissues.

The genes that are targeted during the loss of floral anthocyanins may be constrained by the stress-related functions of anthocyanins and their intermediates. First, anthocyanins are not the only products of the ABP - the early half of the pathway produces several flavonoid intermediates, such as flavonols and flavones, known to function during the plant's stress response [13]. Therefore, if flavonoids are essential for plant survival, loss of function mutations are predicted to persist only in the late genes of the ABP [14]. Second, the loss of anthocyanins may be constrained to ephemeral floral tissues to avoid compromising the vegetative tissue's ability to respond to stress such as exposure to UV, cold, heat, pathogens, or herbivores [12], [15]. Thus, plants that can independently regulate pigment expression in petals and vegetative tissues could respond to selection (pollinator or non-pollinator mediated) acting on petal color while avoiding the deleterious effects of pigment loss in vegetative organs. Decoupling floral and vegetative pigmentation could be accomplished by duplicated genes, each having tissue-specific expression such as the *CHS* duplicates in *Ipomoea purpurea* that are confined to different portions of the corolla [16], [17]. Alternatively, tissue specific expression could be conferred by *cis*- or *trans*-regulatory control of a single locus. For example, the promoter of *CHS* contains binding sites for petal-specific regulatory elements such as bZIP proteins and mybs [18]–[21].

**Figure 2:**
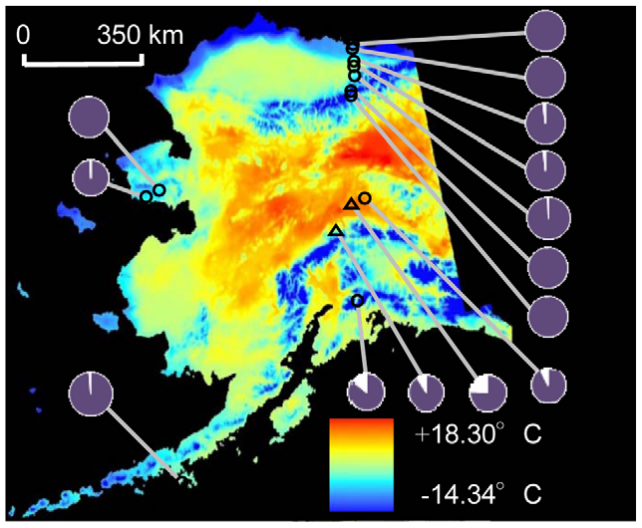
Frequency of *P. nudicaulis* color morphs across Alaska. The proportion of purple- and white-flowered individuals for 14 *P. nudicaulis* populations superimposed on a map depicting the mean July temperature based on historical climate data. The two focal populations used in the phenotypic survey, metabolomic profiling and RNA-based analyses are indicated with triangles (12 Mile Summit and Savage River).

Herein, we have examined the biochemical and molecular basis of a flower color polymorphism in the arctic mustard, *Parrya nudicaulis*. This species exhibits a purple-white flower color polymorphism across its range in Alaska in tundra habitats where pollinators are rare [22], [23] and selection from abiotic factors such as extreme cold, strong winds, the abbreviated growing season and high probability of growing season frost are predicted to be paramount [24], [25]. In *P. nudicaulis*, pollinator visitation rates were not detectably influenced by flower color (Fulkerson and Carlson, unpublished data). Furthermore, the frequency of white-flowered individuals is positively correlated with temperature during the growing season (Figure 2). Our goals in this study are to (1) determine where the ABP is blocked in white petals of *P. nudicaulis* using HPLC and quantitative real-time PCR (qRT-PCR) and (2) establish if the loss of petal anthocyanins also affects leaf pigmentation and gene expression.

## Materials and Methods

### Frequency of flower color morphs across Alaska

The proportion of white- versus purple-flowered individuals was estimated from 14 populations spanning the geographic range of *P. nudicaulis* in Alaska. Populations were chosen based on their geographical distinctiveness and accessibility. All populations were greater than 10 km distant from one another and approximately 250 km apart on average. In order to correlate flower color frequency with climate, we used mean July temperature which approximates the midpoint of the three month growing season for these plants. We also used growing degree days (GDDs) - the annual accumulation of degrees above a minimum baseline temperature below which physiological function is assumed to be inhibited [GDD  =  (maximum temperature – minimum temperature)/2 – baseline temperature]. We compare GDDs calculated using a 4°C baseline with GDDs calculated with a 0°C baseline. All three measures of climate were based on monthly mean temperatures using historic Climate Research Unit data from 1961–1990 and downscaled to a 2 km grid (Nancy Fresco, Scenarios Network for Alaska Planning, University of Alaska Fairbanks). Because of uncertainty in the accuracy of climate predictions at particular geographic localities, we used a Spearman rank-based correlation of climate and flower color.

### HPLC analysis of flavonoids

Although this species shows a wide range of pigmentation levels among purple morphs in all populations, we have focused on only purple- and white-flowered individuals for the following biochemical and molecular analyses. Petal tissue from purple-flowered plants (n = 5) and white-flowered plants (n = 4) was collected from the 12 Mile Summit population (Figure 2) and immediately flash-frozen. Samples were homogenized in 90% HPLC grade methanol (Sigma-Aldrich, St. Louis, MO), centrifuged and the supernatant was run through a Hewlett-Packard Lichrocart 250-4 RP18e 5-µm column on a Hewlett-Packard 1100 series HPLC-DAD instrument. Flavonoids were detected at 210, 254, 330 and 529 nm and were identified by their spectral similarity to known reference compounds [26].

### Phenotypic survey of anthocyanin production across tissue types

A phenotypic survey was conducted at 12 Mile Summit (n = 45) and Savage River (n = 50) populations in June 2008 (Figure 2). Each plant was visually categorized as having either pigmented or non-pigmented petal color. Then, we recorded the presence of pigmentation along the margin of the petiole of the leaves (a common indicator of ABP loss of function in *Arabidopsis thaliana*) and total sepal pigmentation (dark vs. light). Data from both sites were pooled and differences in petal, leaf and sepal tissue pigmentation levels were analyzed using a chi-square test.

### RNA isolation and cDNA synthesis

Tissue from purple- and white-flowered plants was collected from the 12 Mile Summit and Savage River populations (Figure 2). Within a population, three to five individuals with the same petal color were pooled to obtain 50–100 mg of tissue. Petal (n = 12), leaf (n = 13) and sepal (n = 7) tissue was flash-frozen in the field representing a range of developmental stages (petals  =  early bud, late bud, opening, anthesis; leaves  =  young, medium, old). Sepal tissue was pooled across developmental stages since there was no obvious change in pigmentation levels. Tissue was stored at -70°C until RNA could be extracted using the RNeasy Plant Mini Kit (Qiagen, Valencia, CA). We performed a DNaseI treatment on samples using 750 ng RNA and amplification grade DNaseI (Invitrogen, Carlsbad, CA). Purity of RNA was determined with a NanoDrop ND-1000 spectrophotometer (NanoDrop Technologies, Inc., Wilmington, DE) and agarose gels were run to verify RNA integrity. cDNA was synthesized using 750 ng of RNA and the SuperScript III First Strand Synthesis Kit (Invitrogen) with oligo(dT)_20_ primers following the manufacturer's suggested protocol.

### Cloning of ABP genes

We isolated and characterized the six core genes involved in anthocyanin biosynthesis using previously published degenerate primers for angiosperms from Whittall et al. [14]. New degenerate primers were developed for phenylalanine ammonia-lyase (*PAL*). All primer sequences are available on request from the authors. The seven genes were amplified with degenerate primers via reverse-transcriptase polymerase chain reaction (RT-PCR). After gel purification, RT-PCR products were cloned into the pCR 4-TOPO TA vector (Invitrogen). Inserts were confirmed with PCR of the colonies using M13 forward and reverse primers, then sequenced with T3 and T7 primers on an ABI 3730xl DNA Analyzer (Sequetech, Santa Clara, CA) following the suggested BigDye protocol (Applied Biosystems, Foster City, CA). We sequenced an average of 25 clones per gene (range 21–31 clones). Sequences were assembled with Sequencher software (version 4.8; Gene Codes Corporation, Ann Arbor, MI) and aligned with ClustalW [27] in Bioedit (version 7.0.9.0) [28]. The minimum number of recombination events was determined using DnaSP (v. 5.00.07) [29]. Sequence data from cloning and all other sequencing methods have been deposited in GenBank under accession numbers HQ215216-HQ215513.

### Illumina sequencing of arctic mustard transcriptome

To extend the coverage of the ABP genes and recover loci for which there were no degenerate primers available, we compared one early bud sample from each color morph using mRNA-Seq and massively parallel sequencing by synthesis following the manufacturer's protocol (Illumina). On average, we gained 300 bps in the open reading frame (ORF) and 50 bps of UTR sequence (when present) for genes previously characterized with degenerate primers – including 93% of the ORF for *CHS*.

### Genome walking and ABP gene coverage extension

In order to obtain even greater ORF coverage of ABP genes, we designed genome walking primers and amplified 5′ and/or 3′ ends of *PAL*, *CHS*, *CHI*, *F3H*, *DFR* and *ANS* using the manufacturer's suggested protocol (GenomeWalker Universal Kit, Clontech Laboratories, Mountain View, CA). New primers were developed from the genome walking sequences to obtain as much of each gene as possible in cDNA. cDNA from one to two purple-flowered and one to four white-flowered plants was amplified following the methods of Whittall et al. [14].

### Analysis of ABP enzyme-coding genes

We also characterized the flower color genes in genomic DNA to determine whether structural mutations could differentiate populations or color morphs. Samples were collected from purple- and white-flowered plants at eight sites located throughout the geographic range of *P. nudicaulis* (Figure 2) and extracted with the NucleoSpin PlantII kit (Macherey-Nagel, Bethlehem, PA). Primer3 [30] was used to design primers for *PAL*, *CHS*, *CHI*, *ANS* and *UF3GT* that spanned an intron, whenever possible, predicted from the *A. thaliana* genome. We were unable to amplify the *F3H* and *DFR* loci because the location of multiple introns with poly-thymine tracts prevented us from generating reliable sequences. For the other five genes, we amplified 30–40 individuals per gene using crude *Taq* polymerase. Samples were sequenced as described above and the Tajima's *D* statistic was calculated.

### Quantitative Real-Time PCR

Primer and probe sequences for quantitative Real-Time PCR (qRT-PCR) TaqMan assays were designed using Primer Express 3.0 software (Applied Biosystems). Probes were labeled with the 5′ reporter dye 6-FAM and the 3′ quencher Iowa Black FQ. qRT-PCR was performed on the StepOne Real Time PCR System (Applied Biosystems) in 20 µl reaction volumes using the following reagents (and their final concentrations): 1X TaqMan Gene Expression Master Mix, 900 nM forward and reverse primers, 250 nM probe, and 25 ng of cDNA. In addition, 6.25 ng of raw RNA extract was included for five samples and was amplified with each gene to detect the presence of any genomic DNA contamination. Thermal cycling conditions were: 95°C for 10 min, followed by 40 cycles of 95°C for 15 sec and 60°C for 60 sec. Each sample was run in duplicate and the specificity of primers and probes was verified by running the qRT-PCR products on an agarose gel.

Threshold levels for each gene were manually adjusted in order to control for inter-assay variation and relative expression of samples was determined with the comparative ΔΔC_T_ method described in Livak and Schmittgen [31]. The ΔC_T_s for each sample and target were obtained by normalizing the threshold cycle number (C_T_) to the constitutively expressed gene glyceraldehyde-3 phosphate dehydrogenase (GAPDH, amplification efficiency  = 95%). Relative quantification (RQ) values were calculated using the equation RQ  =  (1+E)^−^^ΔΔCT^, where E is the amplification efficiency of each target determined by a standard curve and ΔΔC_T_  =  ΔC_T__purple reference_ – ΔC_T__sample_. The five dilutions used in the standard curve to calculate amplification efficiency were run in triplicate and were serially diluted two-fold from a starting quantity of 20 ng/µl.

Due to limited sample sizes within each developmental stage, samples for each color morph were pooled across developmental stages for statistical analyses. ΔC_T_ values were first converted to a linear form as described in Livak and Schmittgen [31] using the calculation (1+E)^−^^ΔCT^. We compared linear ΔC_T_ values of the two color morphs in each tissue type using a two-sample Student's t-test with unequal sample sizes and unequal variance. To assess significance, we compared our observed t-statistic to t-statistics from 10^4^ bootstrapped datasets of comparable sizes (sampled with replacement).

We tested for correlations among all genes within each tissue type using a Pearson's correlation coefficient. Expression levels for the comparisons also were converted to a linear form as explained above. Data were natural-log transformed to meet assumptions of normality, when necessary, and statistical analyses were completed in Jmp 4.0 (SAS Institute, Cary, North Carolina). In each tissue type, 28 tests were done on all combinations of the eight ABP genes (including FLS, see below). To control for multiple comparisons we used the Bonferroni correction as described in Abdi's equation 8 [32].

### Sequence survey of the *CHS cis* regulatory region

*Cis*-regulation in *CHS* has been well characterized in the Brassicaceae [20] and in many other angiosperms [18], [21], [33]. The locations of the TATA box (−100 bp from start codon) and several petal specific regulatory motifs (within 500 bp of the start codon) in the *CHS* promoter are highly conserved [20]. Therefore, we used genome walking (see methods described above) to characterize 589 bp upstream from the start codon. We then designed *P. nudicaulis*-specific primers for a survey extending 444 bp upstream from the start codon for 10 purple alleles and six white alleles to look for color differentiating SNPs.

### Sequence and expression analysis of *FLS*

Since several candidates for petal specific *trans*-regulation of *CHS* often co-regulate flavonol synthase (*FLS*), we also examined this locus at the sequence and expression levels. Using Illumina transcriptome sequencing, we were able to assemble large portions of *FLS* expressed in petals (841 bps). We amplified *FLS* with cDNA and genomic DNA and designed qRT-PCR assays as for the other ABP genes.

## Results

### Frequency of flower color morphs across Alaska

The percentage of white-flowered individuals among 14 populations ranged from zero to 24% (Figure 2). Five populations had no white-flowered individuals. White flowers were most common in the four interior populations that have higher mean July temperatures (Figure 2) and greater growing-degree days (GDDs), an estimate of the seasonal accumulation of heat available to plants. To test this, we compared the proportion of white-flowered individuals with the number of 4°C GDDs estimated from 30 year historical climate data and found a significantly positive correlation – the proportion of white-flowered individuals decreases as GDDs decrease (Spearman Rank Correlation, rho  = 0.631, *P* = 0.021). The correlation between climate and flower color was equally strong using 0°C as a baseline (rho  = 0.668, *P* = 0.015) or using average July temperature, the midpoint of most populations' three month growing season (rho  = 0.659, *P* = 0.016).

### HPLC analysis of flavonoids

**Figure 3:**
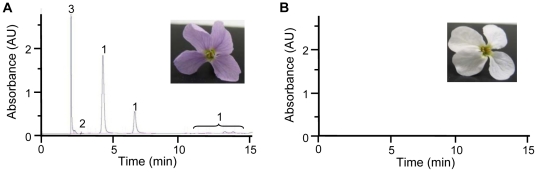
Representative metabolomic profiles of purple and white petals from *P. nudicaulis*. HPLC-DAD detected flavonols (1), catechins (2) and a phenolic acid (3) in purple petals (A). No flavonoid intermediates were detected in white petals (B).

To determine if white flowers were blocked in the early or late genes of the ABP, we compared the metabolomic profiles of petals using methanol extracts on a HPLC equipped with a diode-array detector. Purple petal extracts contained two flavonoids: catechins and flavonols (including both kaempferol and quercetin derived structures), and an unidentified phenolic compound (Figure 3A). Petal extracts of white petals had no detectable catechins and 10^3^× lower flavonol concentrations than purple-petal extracts (Figure 3B). The absence of flavonols and catechins in white petals indicates the ABP is blocked in one of the early genes (*CHS*, *CHI* or *F3H*; Figure 1).

### Phenotypic survey of anthocyanin production across tissue types

We conducted a phenotypic survey to determine if petal color correlated with vegetative pigmentation levels. Ninety-five individuals across two populations were visually scored for flower color, leaf petiole pigmentation and sepal pigmentation. Over 70% of plants had pigmentation on the leaf petiole, regardless of their flower color. There was no significant correlation between flower color and vegetative pigmentation level (χ^2^ = 0.008; df = 93; *P* = 0.93). However, when we compared leaf pigmentation levels with sepal pigmentation, there was a significant correlation (χ^2^ = 4.143; df = 93; *P* = 0.04). Seventy-eight percent of the leaves without visible anthocyanin pigmentation on the petioles had light colored sepals, yet plants with pigmented leaf petioles had an equal frequency of light and dark colored sepals. Although sepals and leaves show correlated pigmentation levels, anthocyanin pigmentation levels in petal and vegetative tissues are decoupled.

### Analysis of ABP enzyme-coding genes

Portions of all seven ABP enzyme-coding genes were examined for DNA sequence changes that could lead to loss-of-function phenotype using RNA samples preserved in the field. A degenerate primer reverse-transcriptase PCR approach [14] combined with sequences derived from genome walking produced an average of 89% of the open reading frame (ORF) for each of the seven ABP enzyme-coding genes (Table 1). We compared these genes for two to three purple-flowered pooled samples (three to five individuals pooled per sample) and two to five similarly pooled white-flowered samples and found the ORF was intact for all seven genes. Each locus harbored an average of 1.8% variation, of which 0.81% was non-synonymous substitutions, among an average of 1157 bp examined (Table 1). There were no consistent sequence differences between white- and purple-flowered samples in enzyme-coding genes. Furthermore, it is doubtful that there are any color differentiating SNPs in the unsampled regions of the ORF or in the adjacent regions since we detected very few recombination events from the degenerate primer reverse-transcriptase PCR approach where we sequenced individual clones and could determine the allelic phase (zero recombination events for *PAL*, *CHI* and *DFR* across an average of 524 bp; 1–2 recombination events for *CHS*, *F3H*, *ANS* and *UF3GT* across an average of 720 bp). The recombination survey was conducted on cDNA sequences within 300 bp of the start and stop codons on average, well within the range of linkage disequilibrium reported from the closely related *Brassica napus* and even *Oryza sativa* [34], [35].

**Table 1:** Summary of variable sites, non-synonymous substitutions, and substitutions per site for ABP loci sequenced from RT-PCR products using degenerate primers and GenomeWalking.

Gene	No. variable sites in ORF	No. non-synonymous substitutions in ORF	No. substitutions per site	Total sequence length(bp)^1^	% ORF
PAL	10	4	0.0048	2085	96
CHS	22	2	0.0186	1185	100
CHI	10	7	0.0166	601	78
F3H	35	12	0.0356	982	83
DFR	17	12	0.0151	1128	100
ANS	23	13	0.0208	1140	97
UF3GT	15	7	0.0153	981	71
FLS	9	1	0.0098	921	91

1 Sequences only contributed to total length and percent open reading frame (ORF) if there was overlap of at least one purple- and one white-flowered individual.

To test whether the same ABP genes were expressed in leaves, we compared petal-derived cDNA sequences to those obtained from leaf cDNAs from purple- and white-flowered plants. Identical alleles were recovered from leaves as were found in the petals for all seven ABP genes, indicating that the same copy was at least weakly expressed in both tissues of both color morphs (RT-PCR conditions optimized for relatively low levels of gene expression).

Since the cDNA comparisons could only include the two populations for which we have preserved RNA samples, we conducted an expanded genomic DNA survey to determine if there were any allele frequency differences in these ABP genes across the species' range in Alaska. We sequenced an average of 52 purple alleles and eight white alleles for an average of 688 bp for all ABP genes except *F3H* and *DFR*, which produced mixed sequence due to poly-T repeats in the introns (Table 2). We found no consistent differences among color morphs in these ABP genes (average coding sequence variation 2.46%, 0.88% of the variation was at non-synonymous sites). For the gene sequences that contained introns (*CHI*, *ANS*, *UF3GT*), intron sequences had higher variation (7.51% variable sites), yet we did not find any consistent differences between color morphs. The lack of distinguishing SNPs in these introns suggests that the immediately adjacent regions of the ORF are unlikely responsible for the purple-to-white transition due to the low frequency of recombination (average distance from intron to start and stop codon is 640 bps). In addition, there were no significant departures from neutrality (Tajima's *D*) in the coding and noncoding regions, when present (Table 2). In summary, all ABP enzyme-coding genes had variable sites, some of which led to amino acid substitutions, yet there was no correlation between structural gene sequence data and flower color.

**Table 2:** Characteristics of the ABP loci used in the genomic DNA survey.

Locus	PCR product length (bp)	Sample size (alleles)	No. variable sites	Coding regionTajima's *D* value^1^	Noncoding region Tajima's *D* value^1^
PAL	657	74	6	−0.21	N/A
CHS	866	64	30	−1.20	N/A
CHI	316	68	9	−0.95	−1.00
ANS	465	66	23	−0.091	−0.24
UF3GT	717	66	25	−1.23	−1.25
FLS	601	60	27	−1.12	−0.27

1 No Tajima's *D* values were significantly different from neutral at the *P*<0.05 level.

### Quantitative Real-Time PCR

**Figure 4:**
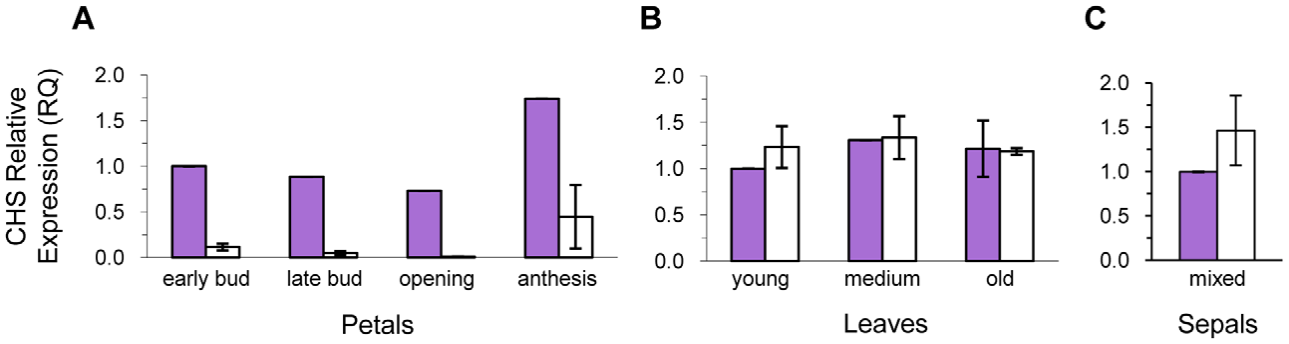
Chalcone synthase expression differences between purple- and white-flowered plants. Expression was measured for petals (A), leaves (B) and sepals (C). Mean relative expression values (± s.e.m. when more than one sample was available) are reported for each color morph across developmental stages in petal and leaf tissue (sepal tissue was pooled across developmental stages). A purple young bud, purple young leaf and purple anthesis sepal served as the reference sample when calculating relative expression. Sample sizes are as follows (purple, white): petals in early bud: N = 1, 2; petals in late bud: N = 1, 3; petals when flowers are opening: N = 1, 1; petals at anthesis: N = 1, 2; young leaves: N = 1, 3; medium leaves: N = 1, 3; old leaves: N = 2, 3; mixed sepals: N = 2, 5.

**Figure 5:**
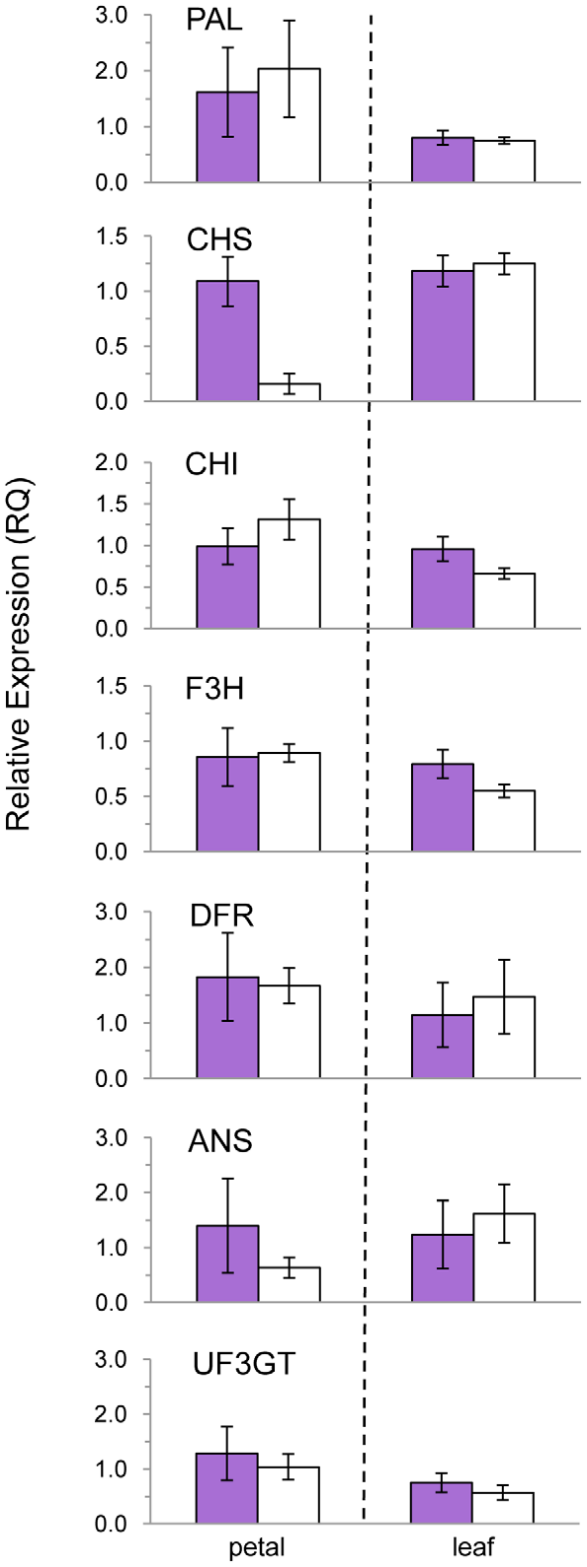
Relative gene expression for each color morph in the anthocyanin biosynthetic pathway. Mean relative expression (± s.e.m.) is shown for petal and leaf tissue, pooled across developmental stages. A purple young bud and purple young leaf served as the reference samples when calculating relative expression. Sample sizes are as follows (purple, white): petal: N = 4, 8; leaf: N = 5, 8.

Gene expression was determined for all seven ABP enzyme-coding genes in both petals and leaves of purple- and white-flowered samples (Table 3). Among petal samples, *CHS* was the only ABP locus where white petals had significantly lower expression than purple petals across developmental stages (average of ∼24×) (Figure 4A; Student's t-test, *P* = 0.0006). The largest difference was at the opening stage, where purple petals had 66× higher expression than white petals. No other genes involved in anthocyanin biosynthesis had significant petal expression differences between color morphs (Figure 5; Table 3). A putative blockage at *CHS*, the threshold of the ABP, is consistent with the absence of flavonols and catechins in white flowers from the metabolomic analysis reported above (Figure 3).

**Table 3:** Effects of flower color on ABP gene expression in petal, leaf, and sepal tissue.

Locus	Amplification Efficiency^1^	Tissue	*P* value^1^	Average RQ (purple/white) ± SE^2^
PAL	0.88449	Petal	N.S.	1.09±3.160
		Leaf	N.S.	1.17±0.181
CHS	1.00000	Petal	0.0006	24.47±0.608
		Leaf	N.S.	0.94±0.525
		Sepal	N.S.	0.68±0.008
CHI	0.83436	Petal	N.S.	0.80±0.485
		Leaf	0.0881	1.57±0.228
F3H	0.92672	Petal	N.S.	0.92±0.321
		Leaf	N.S.	1.69±0.152
DFR	1.00000	Petal	N.S.	1.00±1.225
		Leaf	N.S.	3.04±1.161
ANS	0.91468	Petal	N.S.	14.79±0.192
		Leaf	N.S.	1.56±1.834
UF3GT	0.93975	Petal	N.S.	1.90±0.558
		Leaf	N.S.	1.81±0.284
FLS	0.91181	Petal	N.S.	0.52±0.235
		Leaf	N.S.	1.36±0.235

1 Amplification efficiency for each gene was determined with a standard curve.

2 Average relative expression (RQ) was determined by calculating the gene expression ratio among purple and white flowered individuals at each developmental stage. Ratios were averaged across stages for each tissue type. Sample sizes are as follows: petal: *n* = 12, sepal: *n* = 7, leaf: *n* = 13.

There were no significant differences in expression of ABP genes in leaves from purple- and white-flowered individuals (Figure 5; Table 3). In particular, the range of *CHS* expression in leaves of white-flowered individuals overlapped with that of purple-flowered individuals at all three leaf developmental stages (Figure 4B; Student's t-test *P* = 0.71), consistent with the uncorrelated pigmentation levels of petal and vegetative tissues found in the phenotypic survey described previously. The only gene with a weak, yet non-significant trend in expression differences in leaves of purple and white flowers was *CHI* (purple = 1.6× white; Student's t-test, *P* = 0.088).

Since *CHS* expression was significantly different between purple and white petals, but not between the leaves of those plants, we also examined expression of *CHS* in sepal tissue – the adjacent floral whorl to the petals. Sepals from purple-flowered plants did not have significantly different *CHS* expression than sepals from white-flowered plants (Figure 4C; Student's t-test, *P* = 0.74). In fact, mean *CHS* expression in sepals from white-flowered plants was ∼1.5× higher than that of purple-flowered plants, the opposite pattern detected in the adjacent petals.

Although petal *CHS* was the only locus with differential expression, we looked for correlated expression of ABP loci to test for potential *trans*-acting regulatory elements. We detected significant expression correlations among several of the late genes in the ABP, yet no genes showed a correlation in expression with petal *CHS* (Table 4). In petals, expression of *F3H* and *DFR* was positively correlated (Pearson correlation coefficient *r* = 0.89; *P* = 0.003), as was expression of *ANS* and *UF3GT* (*r* = 0.86; *P* = 0.011). In leaves, expression of *DFR* was correlated with both *ANS* (*r* = 0.90; *P* = 0.0006) and *UF3GT* (*r* = 0.79; *P* = 0.040), yet again, no correlated expression with *CHS* in leaves. Correlated expression of these late genes in the ABP of other angiosperms has often been attributed to one of three conserved regulatory elements, R2R3 MYB, WD40 and bHLH [36], [37], but since petal *CHS* expression does not correlate with any other ABP gene surveyed, the *CHS* downregulation is unlikely to be caused by one of the common ABP regulatory genes.

**Table 4:** Petal and leaf expression correlations among ABP genes^1^.

	PAL	CHS	CHI	F3H	DFR	ANS	UF3GT	FLS
PAL		*r* = 0.584 *P* = NS	*r* = 0.574	*r* = 0.481	*r* = 0.678 *P* = NS	*r* = 0.116	*r* = 0.243	*r* = 0.314
CHS	*r* = 0.384		*r* = 0.152	*r* = −0.09	*r* = 0.125	*r* = 0.128	*r* = −0.065	*r* = 0.053
CHI	*r* = 0.604 *P* = NS	*r* = 0.467		*r* = 0.351	*r* = 0.382	*r* = −0.09	*r* = −0.032	*r* = 0.499
F3H	*r* = 0.516	*r* = 0.239	*r* = 0.669 *P* = NS		*r* = 0.894 *P* = 0.003	*r* = 0.493	*r* = 0.788 *P* = NS	*r* = 0.439
DFR	*r* = 0.192	*r* = −0.09	*r* = 0.301	*r* = −0.15		*r* = 0.456	*r* = 0.740 *P* = NS	*r* = 0.372
ANS	*r* = 0.155	*r* = 0.002	*r* = 0.292	*r* = −0.16	*r* = 0.904 *P* = 0.001		*r* = 0.856 *P* = 0.011	*r* = 0.211
UF3GT	*r* = 0.501	*r* = −0.02	*r* = 0.458	*r* = 0.060	*r* = 0.787 *P* = 0.040	*r* = 0.634 *P* = NS		*r* = 0.288
FLS	*r* = 0.538	*r* = 0.093	*r* = 0.609 *P* = NS	*r* = 0.591 *P* = NS	*r* = −0.21	*r* = −0.33	*r* = 0.339	

1 Values above the diagonal represent correlations in petal expression; values below diagonal are correlations in leaf expression. For each correlation, only Pearson correlation coefficients (*r*) with uncorrected *P*-values <0.05 were tested for true significance using the Bonferroni correction for multiple comparisons. After Bonferroni correction for multiple tests, significant (P<0.05) and non-significant correlations (NS) are indicated.

### Sequence survey of the *CHS cis*-regulatory region

Primers from genome walking produced 444 bp upstream from the start codon. In *CHS*, the 5′ UTR is very short (∼100bp) and we were able to identify the TATA box based on conserved sequence across the Brassicaceae (TTATATA) at 111 bp from the start codon [20]. We identified several petal-specific regulatory motifs upstream from the putative transcriptional start site. We found two H-boxes [aka MRE; CTACC(N)_7_CT] starting 171 and 154 bp upstream from the start codon, comparable to the location of the H-box in Koch et al. [20] and three G-boxes (aka ACE; ACGT) starting 420, 366, and 185 bp from the start codon. In a survey of 10 purple alleles and six white alleles, no SNPs were detected within these motifs and there were no other color differentiating SNPs throughout this relatively short, but critical region of the *CHS* promoter.

### Sequence and expression analysis of *FLS*

In *Arabidopsis thaliana* seedlings, *CHS* expression was correlated with expression of the ABP side-branch enzyme *FLS* via three closely related R2R3-type MYBs (*AtMYB11*, *AtMYB12* and *AtMYB111)* [38]. Similarly, in the white petal margins of *Petunia hybrida* (Baccara Rose Picotee), *CHS* downregulation was also positively correlated with *FLS* expression [39]. Thus, we examined *FLS* for any color-differentiating SNPs and expression differences between purple- and white-flowered individuals. Primers created from Illumina transcriptome sequencing results produced 91.1% of the ORF for *FLS*, yet we did not detect any consistent differences among color morphs in a cDNA survey of two purple- and two white-flowered individuals. Expression analyses of *FLS* showed no significant differences among the color morphs in petal and leaf tissue (Table 3). An expanded genomic DNA survey on a smaller region of the gene (600 bp) also showed no consistent SNPs differentiating purple- and white-flowered individuals in the exons or introns, although there was modest variation (3.9% variation in exons and 5.1% variation in introns; Table 2). Without correlated expression between *CHS* and *FLS*, the probability of *trans*-regulation by the three closely related R2R3-type MYBs causing petal-specific *CHS* differences seems unlikely.

## Discussion

By analyzing flavonoid and RNA samples preserved from subarctic populations of *P. nudicaulis*, we have identified a blockage in *CHS* of white-flowered individuals. The 24× reduction in expression is consistent with the absence of flavonoid intermediates in the petals of white-flowered plants. Studies of floral anthocyanin polymorphisms in natural populations that have found a blockage in *CHS* [40], [41] are less common than reports of later genes in the ABP and transcription factors such as the R2R3 *myb* family controlling late ABP genes [CHS is implicated in only 2/11 pigmented-white transitions summarized in Rausher's recent review [9], [42]]. The bias towards recruitment of late-genes in these examples is presumably due to plant's reliance on essential flavonoid intermediates produced by the early genes (i.e. flavonols) [9], [14], [16]. These protective intermediates accumulate in vegetative tissues of *A. thaliana* following exposure to stresses such as UV-B, cold stress and pathogens often by stimulating *CHS* expression in a light-dependent manner [43]–[47]. Yet, the adaptive benefit of these intermediates and the anthocyanin pigments themselves have been challenging to document [12], [48], [49].

The loss of anthocyanins in petals has been decoupled from the production of vegetative anthocyanins in *P. nudicaulis*. Although the same alleles are expressed in petals and leaves, the significant *CHS* expression difference between purple- and white-flowered individuals is confined to petals, consistent with our phenotypic survey that identified no correlation between petal and vegetative pigmentation. Organ-specific changes in ABP gene expression are not uncommon and likely persist in natural populations since they prevent any deleterious pleiotropic effects of losing flavonoid intermediates and anthocyanin pigments in vegetative tissues [36], [42], [50], [51]. Petal-specific downregulation in the ABP would also prevent more subtle pleiotropic consequences recently associated with floral anthocyanin polymorphisms such as changes in floral scent [52] and decreased leaf glucosinolate induction [6]. In the coldest and harshest growing conditions, we detected a significant reduction in white-flowered individuals, suggesting that the loss of floral anthocyanins and their flavonoid intermediates in petals alone may affect their ability to reproduce in the more extreme climates of arctic Alaska. Transplant experiments along a climatic gradient would be necessary to examine this hypothesis.

The decreased *CHS* expression in white-flowered plants could be caused by *cis-* or *trans-*regulatory changes. *Cis*-regulation caused by mutations in the promoter sequence upstream from the *CHS* transcriptional start site is most consistent with the solitary nature of the *CHS* expression difference among the eight ABP-related loci examined. Furthermore, anecdotal evidence from the Brassicaceae, *Petunia hybrida* and French bean supporting *cis*-regulation of *CHS* has identified petal-specific regulatory motifs in the *CHS* promoter [18], [20], [21], [33], [53] that would allow for the decoupling of petal and vegetative tissue expression in *P. nudicaulis*. Yet, our survey of a portion of the *cis*-regulatory region for *CHS* identified no color-differentiating SNPs. Although we have surveyed the most likely regions immediately upstream from the putative transcriptional start site, we cannot confidently exclude *cis*-regulation until we examine the entire intervening sequence between this gene and the next gene. Since there is no pattern of increasing allelic differentiation as we move upstream from the transcriptional start site, we expect it will take substantial additional sequencing before locating any *cis*-regulatory SNP(s) causing the change in *CHS* expression.

Alternatively, *trans*-regulation through changes in the amino acids of the regulatory element or their expression could lead to the decrease in *CHS* expression in white flowers. *Trans*-regulation of the ABP almost always involves multiple ABP genes [19], [54]. In *P. nudicaulis*, we find no other enzyme-coding genes in the ABP that have correlated expression with *CHS*, although we are able to detect the predicted co-regulation of late genes in the ABP [42]. Furthermore, the most likely candidate for *trans*-regulated *CHS* expression in petals [*Atmyb111;* [38]] also regulates *CHI, F3H,* and *FLS*, yet we find no correlation between *CHS* and these three other genes in *P. nudicaulis*.

In focusing on the loss-of-function phenotypes, in which we expected pleiotropic effects to be most pronounced (pigmented vs. white), we have ignored the variation within the pigmented morphs. Most populations in Alaska exhibit a wide range of variation within the pigmented category ranging from dark purple to light pink. The next step in characterizing this color variation will be to quantify the petal color with UV-Vis spectra, then compare the petal phenotype with their flavonoid profiles and the levels of gene expression across the ABP (especially for *CHS*). Further examination of pigmentation within the flower (i.e. stamens, pollen, and pistil) could also provide additional clues to the target(s) of natural selection on the flower color polymorphism in *P. nudicaulis*. Although the widespread nature of this flower color polymorphism and lack of population structure at neutral loci could facilitate further association mapping, the ability of making controlled crosses has been stymied by the difficulty growing plants under controlled conditions and the length of time it takes them to flower (likely >10 years).

We conclude that white-flowered *P. nudicaulis* are blocked at the threshold to the ABP due to decreased expression of *CHS*. The differential regulation of *CHS* is not present in leaves nor in sepals and therefore, future searches for the selective agents should focus on factors confined to flowers alone. In the Arctic, flower color polymorphisms have been previously attributed to differential heating of the flower [55]. Under sunny conditions, we have detected elevated petal and gynoecium temperature of purple-flowered individuals compared to white individuals of *P. nudicaulis* (Butler, Hesselbach, Carlson and Whittall, unpublished data). Although “thermal rewards” can attract pollinators, since we see no differential visitation to purple and white morphs (Fulkerson and Carlson, unpublished data), we predict the increased floral temperature functions independently of pollinators through expediting fruit maturation by warming the gynoecium - a limiting factor in arctic plant reproduction [55]–[57].
